# Metacognition of Visual Short-Term Memory: Dissociation between Objective and Subjective Components of VSTM

**DOI:** 10.3389/fpsyg.2013.00062

**Published:** 2013-02-14

**Authors:** Silvia Bona, Zaira Cattaneo, Tomaso Vecchi, David Soto, Juha Silvanto

**Affiliations:** ^1^Brain Research Unit, O.V. Lounasmaa Laboratory, School of Science, Aalto UniversityAalto, Finland; ^2^Department of Psychology, University of Milano-BicoccaMilano, Italy; ^3^Brain Connectivity Center, Istituto di Ricovero e Cura a Carattere Scientifico Mondino, University of PaviaPavia, Italy; ^4^Department of Brain and Behavioral Sciences, University of PaviaPavia, Italy; ^5^Department of Medicine, Centre of Neuroscience, Imperial College LondonLondon, UK

**Keywords:** visual short-term memory, visual awareness, visual processing, memory vividness, distracter interference

## Abstract

The relationship between the objective accuracy of visual short-term memory (VSTM) representations and their subjective conscious experience is unknown. We investigated this issue by assessing how the objective and subjective components of VSTM in a delayed cue-target orientation discrimination task are affected by intervening distracters. On each trial, participants were shown a memory cue (a grating), the orientation of which they were asked to hold in memory. On approximately half of the trials, a distracter grating appeared during the maintenance interval; its orientation was either identical to that of the memory cue, or it differed by 10° or 40°. The distracters were masked and presented briefly, so they were only consciously perceived on a subset of trials. At the end of the delay period, a memory test probe was presented, and participants were asked to indicate whether it was tilted to the left or right relative to the memory cue (VSTM accuracy; objective performance). In order to assess subjective metacognition, participants were asked indicate the vividness of their memory for the original memory cue. Finally, participants were asked rate their awareness of the distracter. Results showed that objective VSTM performance was impaired by distracters only when the distracters were very different from the cue, and that this occurred with both subjectively visible and invisible distracters. Subjective metacognition, however, was impaired by distracters of all orientations, but only when these distracters were subjectively invisible. Our results thus indicate that the objective and subjective components of VSTM are to some extent dissociable.

## Introduction

Dissociations between objective and subjective measures of behavior are informative as to the underlying mechanisms of perceptual and cognitive functions. For example, the dissociation between subjective awareness of visual targets and their forced-choice detection accuracy is a hallmark of blindsight, an influential phenomenon in the field of visual awareness (see Cowey, 2010 for review). Consequently, the quality of subjective experiences is increasingly being assessed in conjunction with conventional accuracy measures; response scales such as the Perceptual Awareness Scale (Overgaard et al., 2010; Sandberg et al., 2010) have been developed for this purpose. These scales can assess the so-called metacognitive sensitivity by revealing how well subjective ratings correlate with performance on objective detection or discrimination tasks (e.g., Lau and Passingham, 2006).

In research on visual short-term memory (VSTM), the relationship between the objective and subjective components of memory representations has not been investigated. Recently, it has been shown that working memory processes can operate unintentionally outside of conscious awareness (Hassin et al., 2009), as well as on subliminally presented stimuli (Soto et al., 2011), indicating that VSTM processes can be dissociated from conscious experience. However, the relationship between the *subjective experience of the memory representation* and its objective accuracy has not been investigated. These two aspects are conflated in standard VSTM experiments, which involve both objective and subjective components, but assess only the former (i.e., VSTM accuracy). The aim of the present study was to assess and compare these objective/subjective components by determining their susceptibility to visual distracters presented during VSTM maintenance. Subjective vividness of the VSTM content was measured on a trial-to-trial basis in addition to assessing objective VSTM accuracy. We used a paradigm known as “memory masking” (Magnussen et al., 1991; Magnussen, 2009), in which a distracter stimulus is presented briefly during the interval between the memory cue and test. We predicted that objective VSTM ought to be impaired when the distracter orientation is sufficiently different from the memory cue (Silvanto and Soto, 2012). The critical question is whether or not the subjective vividness component of VSTM would follow the same pattern.

## Materials and Methods

### Participants

Seventeen participants who were naïve to the aims of the study, took part in the experiment; of those three were excluded due to chance level performance in the VSTM task; thus 14 participants were included in the data analyses (six males, mean age 24 years). All participants provided informed consent and were given a monetary reward for their participation.

### Stimuli and experimental procedure

**Figure 1:**
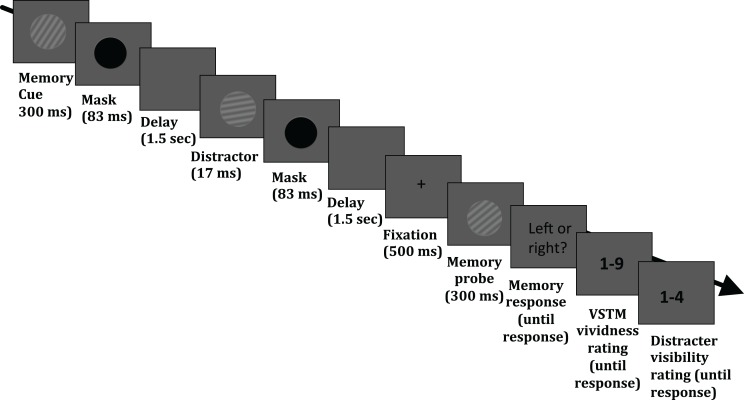
**Timeline of an experimental trial**. Participants were asked to hold in memory the orientation of a grating; at the end of each trial, they performed an orientation discrimination task (indicating whether a test probe was tilted to the left or right relative to the memory cue). On half of the trials, a visual distracter (a grating with the same size, contrast and spatial frequency as the memory cue) was presented in the middle of the delay period; the orientation of this distracter could be identical to that of the memory cue, or it could differ by 10° or 40°. In addition, participants were asked to rate the subjective vividness of their memory representation as it was at the end of the delay period (“How vivid was the memory item in your memory?”), on a 1–9 scale, in which 1 means the absence of a mental image and 9 refers to an image that is as clear and vivid as visual perception of the grating. Finally, observers were asked to indicate whether they had perceived the visual distracter appearing during the maintenance period, using a 4-point scale, adapted from Overgaard et al. (2010) and Sandberg et al. (2010): 1 = did not see the distracter; 2 = maybe saw something; 3 = saw the distracter but not its orientation; 4 = saw the distracter and its orientation.

The stimuli were presented on a 19″ (1280 × 1024 pixels) CRT monitor with a refresh rate of 60 Hz. Stimuli and task were controlled by E-Prime v2.0 (Psychology Software Tools Inc., Pittsburgh, USA; http://www.pstnet.com/eprime.cfm). The task required the maintenance of a sinusoidal luminance-modulated grating in VSTM while a masked distracter grating was presented, on half of the trials, during the 3.1 s delay period (see Figure 1). Each trial began with a black fixation point appearing in the middle of the screen for 1000 ms, followed by a blank screen for 500 ms. The memory cue (orientation 10°, 20°, 30°, 40°, or 50° to the left or right from vertical; 0.1 Michelson contrast; spatial frequency 1 cycle/°; diameter 4° of visual angle from a viewing distance of 72 cm) was then presented for 300 ms, followed by a mask (a black circle presented for 83 ms). On 50% of trials, a distracter was presented 1.5 s after the mask offset for 17 ms and its orientation was either the same as that of the memory item, or it differed by 10° or 40°. The spatial frequency, contrast, size, phase, and location were the same as that of the memory cue. On half of the trials, no distracter was presented. The distracter was followed by a mask (83 ms), which was presented on all trials, even when no distracter was presented. The average brightness of the background was the average brightness of the gratings. At the end of the maintenance period, fixation (500 ms) was presented, followed by the memory test probe (tilted 10° either to the left or right relative to the memory cue) and observers had to indicate (during an unlimited time window) whether the test probe was tilted to the left or right relative to the memory cue. This was indicated with a button press (“1” for leftwards and “2” for rightwards). After this response had been given, a screen asking participants to rate their subjective vividness of their memory representation appeared; subjects were instructed that they should report vividness as it was at the end of the delay period (the specific instruction was “How vivid was the memory item in your memory?”), on a 1–9 scale similar to used in studies on visual imagery (e.g., Baddeley and Andrade, 2001), in which 1 means the absence of a mental image and 9 refers to an image that is as clear and vivid as visual perception of the grating. Finally, after the vividness response had been given, a screen prompting participants to report whether they had perceived the visual distracter appearing during the maintenance period, using a 4-point scale, adapted from Overgaard et al. (2010) and Sandberg et al. (2010): 1 = did not see the distracter; 2 = maybe saw something; 3 = saw the distracter but not its orientation; 4 = saw the distracter and its orientation. The response windows had no time limit. A blank screen (duration 1 s) was presented before the next trial commenced.

The combination of memory cue orientation (either 10°, 20°, 30°, 40°, or 50°); distracter condition (No Distracter, 0° difference relative to the memory cue, 10° difference, or 40° difference) and correct response in the memory task (left or right) produced a combination of 40 different trial types. The experiment was run in six blocks of 80 trials. The trial selection was fully randomized, such that each trial was randomly chosen from all possible trial types, with the weight of “No Distracter” trials being the same as that of all “Distracter” present trials combined (i.e., the likelihood of a No Distracter trial was set to be 50%). The mean total number of trials for each of the condition was: No Distracter condition = 229 trials; 0° distracter condition: 84 trials; 10° distracter condition: 84 trials: 40° distracter condition: 82 trials.

## Results

**Figure 2:**
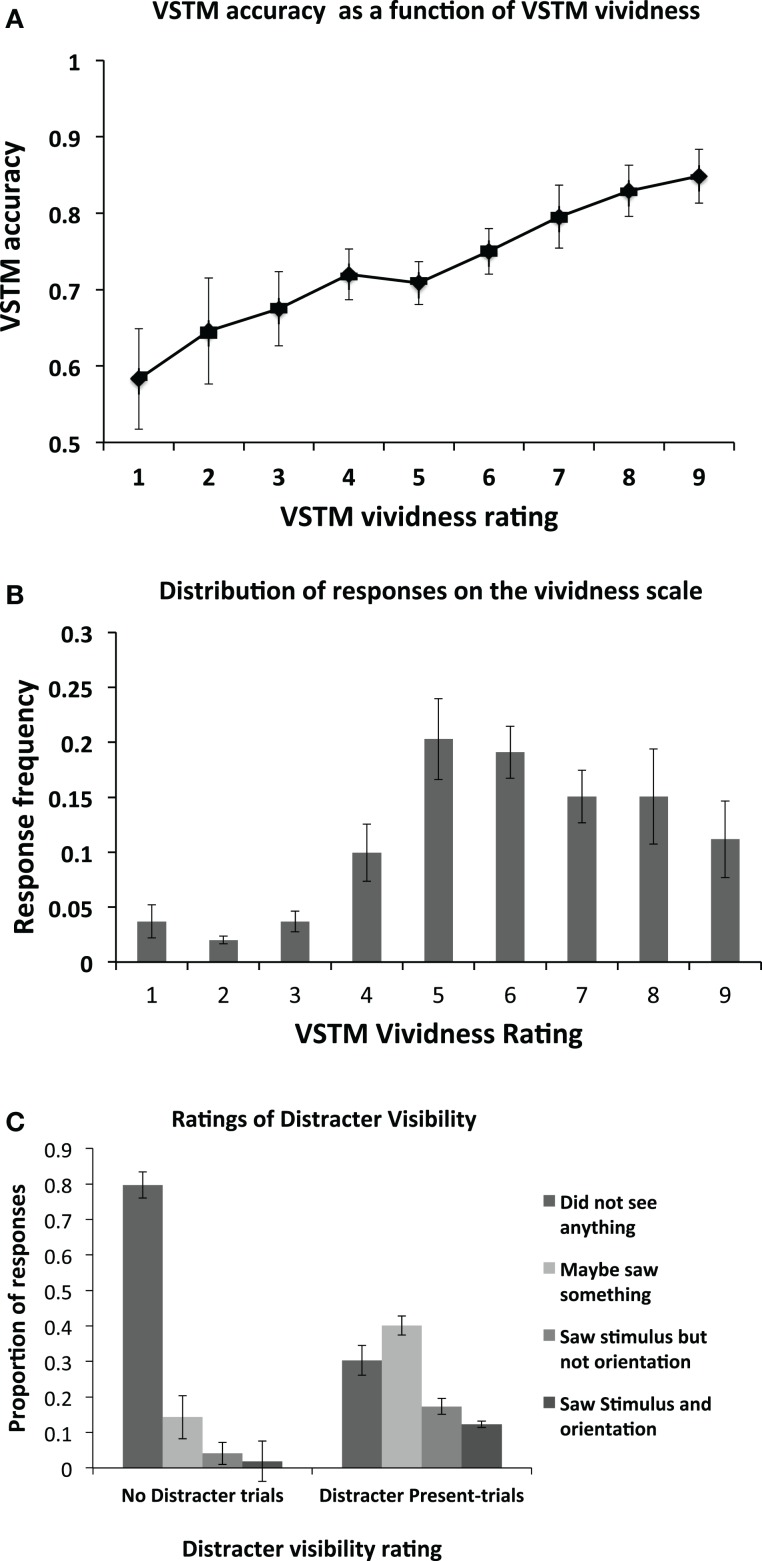
**(A)** Mean (*n* = 14) objective VSTM accuracy as a function of subjective VSTM vividness. On each trial, in addition to performing the objective VSTM discrimination task, participants were asked to provide a rating of the subjective vividness of their memory representation using a 1–9 scale. As expected, there was a positive correlation between these measures, with high VSTM accuracy associated with high levels of subjective vividness. This correlation was statistically significant (Pearson’s *r* = 0.459; *p* < 0.01). Error bars indicate ±1 SEM. **(B)** The distribution of participants’ responses on the vividness scale. On each trial, participants were asked to indicate the subjective vividness of their memory representation on a scale ranging from 1 to 9. The figure indicates the proportion of responses for a given category of vividness. **(C)** Proportion of visibility responses for the masked visual distracter presented during the maintenance period for the No Distracter- and Distracter-Present conditions.

We first assessed the relationship between the VSTM accuracy and vividness, independently of any effect induced by the distracters; this is shown in Figure 2A. As expected, there is a positive correlation between the two, with high VSTM performance associated with high levels of vividness. This correlation was statistically significant (Pearson’s *r* = 0.459; *p* < 0.01). The mean frequency of responses at each level of the vividness scale is shown in Figure 2B. Proportion of visibility responses for the visual distracter presented during the maintenance period is shown in Figure 2C. We additionally used signal detection theory to derive a measure of perceptual sensitivity for the distracter. A hit was defined responses where participants reported occurrence of the distracter (i.e., responses 2, 3, 4) when it was actually present (i.e., distracter-present trials). False alarms were defined as the same responses on trials where the distracter was absent (i.e., on “No Distracter-trials”). The mean *d*’ score was 1.52 (SD = 0.61), and the criterion 0.183 (SD = 0.37). This *d*’ value indicates that participants were generally able to detect the distracter at a level which is clearly above chance, indicating that the mask did not render the distracter fully invisible. The criterion value being close to 0 indicates that the participants had no consistent bias toward reporting either distracter presence on “No distracter”-trials or distracter absence on “distracter-present” trials.

We then divided the trials according to distracter visibility. Trials on which subjects reported full unawareness of the distracter, i.e., response “1” on the scale of Overgaard et al., 2010; “did not see the distracter”) were classified as “reported unawareness.” This corresponded to on average 31% (SD = 14) of distracter-present trials. The trials where participants correctly indicated awareness of the distracter (i.e., responses 2–4 on the scale of Overgaard et al., 2010) were classified as “reported awareness”. When no distracter was presented, participants reported unawareness (i.e., response “1”) of the distracter on average 79% (SD = 16) of trials. Only these trials are included in the “No Distracter” condition in the analyses below (i.e., false alarms were not included).

### Impact of distracter visibility and orientation on VSTM accuracy

**Figure 3:**
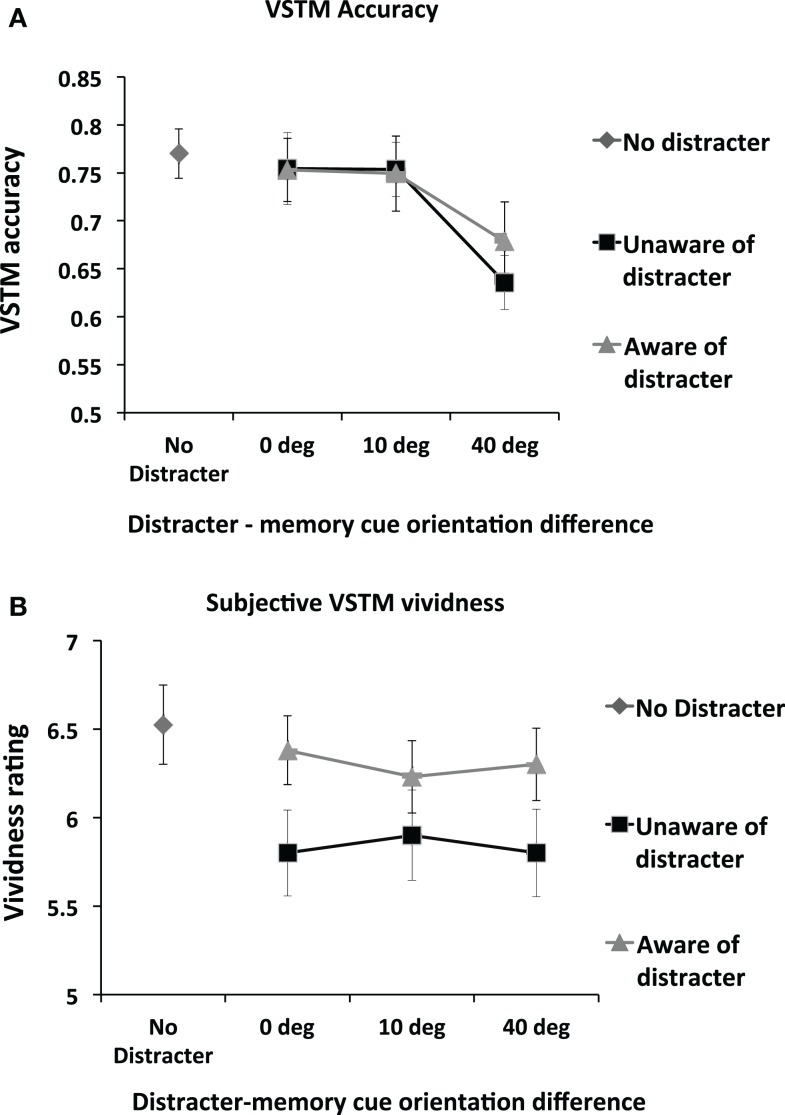
**(A)** Mean (*n* = 14) objective memory accuracy in the VSTM task as a function of the distracter orientation and visibility. The impact of the visual distracter on VSTM accuracy was determined by the orientation difference between the memory cue and the distracter; a reduction in accuracy was observed only when this difference was 40°. Distracter visibility did not influence this effect; a disruptive effect was observed regardless of whether participants rated the distracter to be visible. Error bars indicate ±1 SEM. **(B)** Mean (*n* = 14) subjective VSTM vividness as a function of the distracter orientation and visibility. The impact of the visual distracter on subjective vividness of the memory representation was not modulated by the orientation of the distracter. Rather, all distracter orientations reduced vividness ratings, but only when the visual distracter was judged not to be visible. Thus a double dissociation in the effects of distracters on VSTM accuracy and subjective vividness was found: the impact on VSTM accuracy depends on the orientation difference between the memory cue and the distracter but not distracter visibility; the impact on subjective vividness of the memory item was determined by distracter visibility but not by orientation difference.

Figure 3A shows VSTM accuracy as a function of distracter visibility and orientation. We first assessed the impact of distracter visibility and orientation on the accuracy of VSTM by means of repeated-measures ANOVA with distracter visibility (subliminal; visible distracter) and orientation difference relative to memory cue (0°, 10°, 40°) as main factors. (This ANOVA included only distracter-present trials; distracter-absent trials were not included.) This revealed a significant effect of orientation difference [*F*(2,26) = 10.87; *p* = 0.0001; partial η^2^ = 0.455] but no main effect of distracter visibility [*F*(1,13) = 0.43; *p* = 0.53] and no interaction [*F*(2,26) = 0.71; *p* = 0.50]. Pairwise comparisons on the factor “orientation difference” (collapsing over the non-significant factor “visibility”) revealed that, relative to all other conditions, memory accuracy was reduced when distracter orientation differed from the memory cue by 40° [*vs. 0*°*distracter*: *t*(13) = 3.55; *p* = 0.004; *vs*. *10*°*distracter:* *t*(13) = 4.32; *p* = 0.001]. No other significant effects were found.

We carried out a separate set of *t*-tests to compare the impact of the distracter relative to the No Distracter condition. A significant reduction in accuracy was observed relative to the No Distracter condition in both the visible [*t*(13) = 3.08; *p* = 0.009] and invisible [*t*(13) = 4.02; *p* = 0.001] conditions for the 40° condition. No other significant effects were found. In short, VSTM accuracy was reduced when the distracter differed from the memory cue by 40°, regardless of whether the distracter was consciously perceived.

### Impact of distracter visibility and orientation on subjective VSTM vividness

Figure 3B shows subjective VSTM vividness as a function of distracter visibility and orientation. We first assessed the impact of distracter visibility and orientation on vividness by means of repeated-measures ANOVA with distracter visibility (subliminal; visible distracter) and orientation difference relative to memory cue (0°; 10°; 40°) as main factors. (This ANOVA included only distracter-present trials; distracter-absent trials were not included). A significant main effect of visibility was found [*F*(1,13) = 4.97; *p* = 0.04; partial η^2^ = 0.277], with subjective vividness being significantly worse in the subliminal condition. However, neither a main effect of distracter orientation [*F*(2,26) = 0.11; *p* = 0.90] nor an interaction of visibility by distracter [*F*(2,26) = 1.89; *p* = 0.17] was found.

We carried out a separate set of *t*-tests to compare the impact of the distracter relative to the No Distracter condition. Relative to the No Distracter condition, a significant reduction in vividness was observed for all subliminal distracters [*0*°*:* *t*(13) = 5.32; *p* = 0.0001; *10*°*:* *t*(13) = 3.34; *p* = 0.005; *40*°*:* *t*(13) = 4.15; *p* = 0.001] but not for any of the visible distracters [*0*°*:* *t*(13) = 0.90; *p* = 0.38; *10*°*:* *t*(13) = 1.68; *p* = 0.12; *40*°*:* *t*(13) = 1.19; *p* = 0.26]. Thus subjective VSTM vividness was reduced by distracters of all orientations when participants reported unawareness of them. In contrast, distracters that participants reported to be aware of had no significant impact.

We also assessed whether performance on Distracter-absent trials on which participants reported the presence of a distracter (i.e., false alarms) differed from trials on which participants indicated correctly the absence of the distracter (i.e., correct rejections). There was no difference between these trials types for neither VSTM accuracy [*t*(13) = 0.03; *p* = 0.97] nor subjective vividness [*t*(13) = 1.60; *p* = 0.13]. The mean VSTM accuracies were 0.77 (SD = 0.17) and 0.77 (SD = 0.10) for false alarm and correct rejection trials, respectively. For vividness, the mean ratings were 6.15 (SD = 1.20) and 6.50 (SD = 0.84) for false alarm and correct rejection trials, respectively.

## Discussion

The aim of this study was to investigate the relationship between objective and subjective components of VSTM by assessing their susceptibility to distracting visual information. For VSTM accuracy, interference was observed when the orientation of the distracter differed from the memory cue by 40°; no effect was found when they differed by 0 or 10°. The effect on accuracy was not modulated by cue visibility. In contrast, VSTM vividness was impaired by distracters of all orientations, but only when participants reported unawareness of them. That the objective and subjective components of VSTM are differentially susceptible to distracting visual input provides the first evidence (to the best of our knowledge) that their underlying mechanisms are to at least some extent distinct. These results cannot simply be due to either VSTM accuracy or vividness being more susceptible to distracters, as a double dissociation in the effects of distracter visibility and orientation was observed. It is important to note that overall there was a clear positive correlation between VSTM accuracy and its subjective vividness, indicative of high metacognitive sensitivity. In other words, subjective vividness ratings generally reflected the accuracy of short-term memory.

Our results on VSTM accuracy of orientation information fit well with previous memory masking literature on how visual distracters affect the accuracy of representations held in VSTM (Magnussen et al., 1991; Magnussen, 2009). In these previous studies, participants’ ability to maintain the spatial frequency of the memory cue was assessed; the disruptive effect of the distracter increased as its spatial frequency moves away from the memory frequency; no masking effect was found when the spatial frequency of the memory and distracter gratings were similar (e.g., Magnussen et al., 1991). The largest impairment was found at a difference of ±1 octave, the width of spatial frequency channels reported in psychophysical studies (Blakemore and Campbell, 1969; Greenlee and Magnussen, 1988). Thus the effects reported by Magnussen and colleagues are likely to reflect competition between spatial frequency channels. Our study mirrors these results in the domain of VSTM for orientation information: a disruptive effect on VSTM accuracy was found when the distracter differed from the memory item by 40°, indicative of competition between orientation-selective channels, the width of which is believed to be in the range of 30°–40° (e.g., Blakemore and Campbell, 1969; Campbell and Maffei, 1971; Greenlee and Magnussen, 1988). In short, the present result on accuracy may reflect competition between orientation-selective channels, mirroring previous studies on spatial frequency (Magnussen et al., 1991).

In contrast, VSTM vividness was reduced by all distracter orientations, but only when participants reported being fully unaware of them. A simple speculative explanation is that the engagement of the visual system by visual input removes attentional resources from the subjective mental image. Subliminal distracters may be more effective in contaminating subjective experience as they are less likely to be inhibited by top-down control (Tsushima et al., 2006). Furthermore, conscious detection of the distracter may have induced participants to allocate more attentional resources to the memory representation, counteracting any disruptive effect of the distracter. Such a strategic response cannot occur if the distracter is not consciously perceived. This finding is also consistent with the evidence that static visual noise of which participants are aware does not impair visual imagery (Quinn and McConnell, 1996, 2006; see also next paragraph). While the relationship between memory vividness and distracter awareness requires further study, the key finding here is that effect on subjective VSTM vividness was not modulated by distracter orientation. This indicates that the subjective component of VSTM is not merely an extension of the underlying VSTM representation. Rather, the objective and subjective components appear to be to some extent distinct. It may be that the subjective experience of VSTM representations requires an active processing “workspace” that is separate from a passive VSTM store. Such a possibility was suggested by Pearson et al. (1999), who proposed a distinction between an active visual buffer for conscious visual representations, and a passive visual cache for unconscious storage of visual representations. While the precise nature of the objective vs. subjective distinction requires further study, the nature of interference effects observed in the present study indicates that the objective VSTM store relies on feature-specific mechanisms (such as orientation channels) in the visual system, whereas its subjective component does not.

These results could be seen to relate to the literature on visual imagery, assuming that the subjective component of VSTM can be equated with the subjective experience of mental images in conventional imagery paradigms. Prior studies have used dynamic visual noise (DVN) to investigate the cognitive structures underlying VSTM and imagery (Quinn and McConnell, 1996, 2006). In these studies, the presentation of DVN has been shown to reduce the ability to engage in mental imagery; in contrast, presentation of static visual noise has no impact. The present work differs from this line of research in a number of ways. Firstly, here we addressed both VSTM accuracy and subjective VSTM vividness on a trial-to-trial basis, enabling a direct comparison of the objective and subjective components of the same stimulus. Such direct comparisons, to the best of our knowledge, have previously not been carried out. Secondly, the DVN is silent as to how the features and visibility of distracting information modulate imagery and VSTM; the present paradigm dissociates these aspects. Thirdly, the impact of stimulus visibility has not been previously addressed; in the DVN paradigm, the noise is always consciously perceived. It is worth noting that the presentation of static visual noise has no impact on visual imagery; our result that consciously perceived visual distracters had no impact on subjective vividness is consistent with this.

A comparison of objective and subjective measures can raise the question of the extent to which the obtained dissociations are driven by differences in sensitivity between the measures. One could argue that in the present study, objective accuracy may have been less sensitive to the effect of distracter orientation because vividness was measured on a 9-point scale, whereas accuracy was measured by a binary (left/right) decision; perhaps a more sensitive scale might have revealed effects on accuracy at other distracter orientation (as was found for vividness). However, this explanation is not consistent with the finding that objective accuracy and subjective vividness showed a double dissociation in their sensitivity to different features of distracters (orientation and visibility), rather than vividness being generally more susceptible to disruption. If it was indeed the case that vividness was the more vulnerable of the two measures, then one would not have observed a selective effect of distracter visibility on vividness ratings (in which only subliminal distracters reduced vividness), given that VSTM accuracy was impaired by both subliminal and visible distracters, as long as there was a large enough difference (40°) between the orientations of the memory cue and distracter.

Visual short-term memory accuracy and subjective vividness on Distracter-absent trials on which participants reported the presence of a distracter (i.e., false alarms) did not differ significantly relative to trials on which participants indicated correctly distracter absence (i.e., correct rejections). This is consistent with the main results of the study, as VSTM accuracy was impaired only by specific distracter orientations, and vividness was significantly reduced only by stimuli of which participants were unaware. One should note also that the effects reported here might be modulated by participants’ criterion for reporting stimulus presence, as this might alter the effect of unaware vs. visible distracters on subjective vividness. Future studies manipulating the proportion of distracter-present and distracter-absent trials are required to determine this issue.

In summary, these results provide the first evidence that objective and subjective components of VSTM are to some extent dissociable. What are these mechanisms? While the present results do not offer a conclusive explanation, one could speculate the following. That the impact of the distracter on VSTM accuracy depended on the similarity between memory cue and distracter orientation indicates that the WM representation makes use of orientation channels in the visual cortex (e.g., Blakemore and Campbell, 1969; Campbell and Maffei, 1971); subjective vividness was not sensitive to orientation similarity, indicating it does not reflect the activity of orientation channels. In contrast, subjective vividness was sensitive to the visibility of the distracter, consistent with the evidence that subliminal distracters are more difficult to suppress, implicating prefrontal attentional control mechanisms (Tsushima et al., 2006; Feredoes et al., 2011). VSTM accuracy was not sensitive to distracter visibility, consistent with the idea that it engages low-level orientation channels in the visual system which operate independently of visual awareness. It should be noted that this explanation is somewhat speculative and requires further study.

## Conflict of Interest Statement

The authors declare that the research was conducted in the absence of any commercial or financial relationships that could be construed as a potential conflict of interest.
